# Comparative Analysis of Mare Milk and Foal Faecal Microbiota in Kyrgyz Mare–Foal Pairs During the First 21 Days Postpartum

**DOI:** 10.3390/ani16213448

**Published:** 2026-11-01

**Authors:** Tarik Safak, Elena Stancheva, Toncho Penev, Hafize Tuğba Yüksel-Dolgun, Ali Risvanli, Oznur Yılmaz-Koç, Özgül Gülaydın, Ruslan Salykov, Ömer Deniz, Özgür Kaynar, Oğuzhan Dolgun

**Affiliations:** 1 Department of Obstetrics and Gynecology, Faculty of Veterinary Medicine, Kastamonu University, 37000 Kastamonu, Türkiye; 2 Department of Ecology and Animal Hygiene, Faculty of Agriculture, Trakia University, 6000 Stara Zagora, Bulgaria; elena.stancheva@trakia-uni.bg; 3 Department of Microbiology, Faculty of Veterinary Medicine, Aydın Adnan Menderes University, 09010 Aydın, Türkiye; tugba.yuksel@adu.edu.tr; 4 Faculty of Veterinary Medicine, Kyrgyz Turkish University, Bishkek 720044, Kyrgyzstan; arisvanli@firat.edu.tr (A.R.); ruslan.salykov@manas.edu.kg (R.S.); 5 Department of Obstetrics and Gynecology, Faculty of Veterinary Medicine, Fırat University, 23270 Elazıg, Türkiye; 6 Department of Obstetrics and Gynecology, Faculty of Veterinary Medicine, Siirt University, 56100 Siirt, Türkiye; oznur.yilmaz@siirt.edu.tr; 7 Department of Microbiology, Faculty of Veterinary Medicine, Siirt University, 56100 Siirt, Türkiye; ozgul.gulaydin@siirt.edu.tr; 8 Department of Internal Medicine, Faculty of Veterinary Medicine, Kastamonu University, 37000 Kastamonu, Türkiye; odeniz@kastamonu.edu.tr; 9 Department of Biochemistry, Faculty of Veterinary Medicine, Kastamonu University, 37000 Kastamonu, Türkiye; ozgurkaynar@gmail.com; 10 Department of Veterinary Microbiology, Institute of Health Sciences, Aydın Adnan Menderes University, 09010 Aydın, Türkiye; oguzhandolgun@gmail.com

**Keywords:** faecal microbiota, foal, mare, milk microbiota

## Abstract

This pilot study characterised bacterial DNA profiles in 40 milk and 39 faecal samples from eight Kyrgyz mare–foal pairs collected at birth, 24 h, and days 7, 14, and 21 postpartum using V3–V4 16S rRNA gene sequencing. Permutation Friedman tests detected temporal differences in foal faecal Shannon diversity and observed feature counts (Holm-adjusted *p* = 0.020 for both), but not in milk. Faecal diversity was descriptively lower at 24 h and day 7 than at birth and increased at days 14–21. Animal-blocked weighted UniFrac PERMANOVA detected a temporal effect in faecal DNA profiles (Holm-adjusted *p* = 0.0028), but not in milk (*p* = 0.0956). Pair-blocked milk–faecal comparisons were significant at each time point (Holm-adjusted *p* = 0.0391), although differences in dispersion limit interpretation. Without sequenced negative controls, background contamination cannot be distinguished from genuine milk-associated bacterial DNA; therefore, milk profiles cannot be confidently attributed to resident milk microbiota. Observed taxonomic similarities do not demonstrate microbial transmission or milk-derived foal gut colonisation. Significance in faeces but not milk does not establish a difference between their temporal responses, which was not directly tested. These findings are exploratory and require controlled validation.

## Simple Summary

Understanding how bacterial profiles change in mare milk and foal faecal samples during early life may help guide research on foal health and management. We examined 79 samples collected from eight Kyrgyz mare–foal pairs at birth, 24 h, and 7, 14, and 21 days postpartum. Statistically significant temporal changes were detected in foal faecal bacterial DNA profiles, but not in milk profiles. However, this does not establish a significant difference in temporal responses between the two sample types, as no direct interaction test was performed. Faecal bacterial diversity was not lowest at birth: median values decreased at 24 h and 7 days and subsequently increased at 14–21 days. Although milk and faecal samples shared some bacterial groups, this does not demonstrate bacterial transfer through milk. The small number of animals and absence of sequenced negative controls limit interpretation, particularly for milk samples, in which background contamination could influence the detected profiles. These findings are exploratory and require confirmation in studies with appropriate controls.

## 1. Introduction

The mare’s epitheliochorial placenta prevents the prenatal transfer of maternal antibodies to the foal. Consequently, foals are born physiologically hypogammaglobulinemic as a direct result of this placental structure [1,2]. Although the immune organs of neonatal foals are morphologically developed at birth, the effective immune response required for protection develops only after approximately two weeks because of this hypogammaglobulinemia state. During this early postnatal period, foals are highly susceptible to infections, making the prompt ingestion of colostrum immediately after birth critically important [3]. Colostrum contains numerous components that are essential for the establishment of neonatal immunity and intestinal maturation in the foal [4]. Mare colostrum is a highly nutritious secretion rich in biologically active substances that play a critical role in protecting foals against infections. These components include, most notably, immunoglobulins, as well as proteins, vitamins, minerals, antimicrobial peptides, lactoferrin, growth factors, lysozyme and hormones [5]. As in the neonates of other species [6], the Brix refractometer (BRM) is used in horses to estimate Immunoglobulin G (IgG) concentrations. The BRM is also commonly applied on equine breeding farms to assess colostrum quality [7]. Using the BRM, colostrum quality is evaluated primarily with respect to its IgG content. According to BRM measurements, colostrum quality is classified as poor (0–15% Brix), borderline (15–20% Brix), adequate (20–30% Brix), and very good (>30% Brix) [8]. Accordingly, measurement of serum IgG concentration is used to determine the level of maternally derived passive immunity in foals [9]. Failure of passive transfer (FPT) occurs when neonatal foals do not receive enough high-quality IgG, with reported prevalence ranging from 3% to 37.8%. In foals, FPT is also classified based on serum IgG concentrations: values < 400 mg/dL indicate complete FPT, whereas concentrations between 400 and 800 mg/dL are considered partial FPT [10,11].

Longitudinal studies have documented age-associated changes in foal faecal microbiota during postnatal development [12,13]. Direct comparisons of mare milk and foal intestinal bacterial profiles provide a complementary perspective on this process. Mienaltowski et al. [14] followed 15 mare–foal pairs over the first 15 days postpartum and reported increasing differentiation between milk and foal rectal-swab communities, suggesting that sources other than milk contribute substantially to early gut microbial development. Building on this work, the present pilot study examines paired mare milk and foal faecal bacterial DNA profiles in a Kyrgyz cohort, including sampling immediately after birth (0 h) and follow-up through day 21. Its contribution is an exploratory description of these profiles across the immediate neonatal period and the third postnatal week, rather than evidence of microbial transmission or a comprehensive characterisation of colonisation sources.

This pilot study aimed to characterise bacterial DNA profiles detected in mare colostrum and milk samples at 0 h, 24 h, and days 7, 14, and 21 postpartum using V3–V4 16S rRNA gene amplicon sequencing. In addition, changes in the faecal microbial composition of neonatal foals born to and suckling from these mares were evaluated at the same postpartum time points (0 h, 24 h, and days 7, 14, and 21).

## 2. Materials and Methods

### 2.1. Animals and Housing

The study was conducted at a privately owned facility providing mare breeding and boarding services and involved eight Kyrgyz breed mares and their foals. This study included eight healthy Kyrgyz breed adult pregnant mares (average age: 6 years). The body weights of the mares were estimated to range from approximately 300 to 350 kg, while the birth weights of the foals included in the study ranged from approximately 25 to 30 kg. All foals used in the study were sired by a single stallion of the same breed. Mares and foals presenting with systemic disease, diarrhea, metritis, mastitis, or any condition requiring medical treatment (e.g., antibiotic administration), as well as those requiring surgical intervention, were excluded from the study. Of the 29 pregnant Kyrgyz mares on the farm, only eight met the inclusion criteria and were enrolled; all had normal vaginal parturition, and mares experiencing dystocia were excluded. In addition, only female foals were included to establish a more homogeneous study population and minimize potential sex-related variability. All eight foals included in the study were female.

Kyrgyz mares in the enterprise were fed according to a defined ration. The concentrate feed was formulated to contain 44% rolled oats, 24% crushed barley, 12% maize, 15% soybean meal, 4% wheat bran, 0.5% salt, and 0.5% toxyfix (Veritasvet, Germany). During gestation, mares received a total of 4 kg concentrate feed per day (2 kg in the morning and 2 kg in the evening), in addition to 4 kg hay (2 kg morning, 2 kg evening) and 3 kg alfalfa (1.5 kg morning, 1.5 kg evening). Following parturition, the same ration composition was maintained; however, feeding levels were adjusted such that mares were provided with 6 kg concentrate feed per day (3 kg morning, 3 kg evening), 5 kg hay (2.5 kg morning, 2.5 kg evening), and 4 kg alfalfa (2 kg morning, 2 kg evening) [15].

### 2.2. Study Groups and Samples

Colostrum and milk samples were collected from mares that had completed gestation and delivered normally. Before suckling, colostrum quality was evaluated using a BRM [8]. Mares were divided into two groups based on colostral Brix readings; Kyrgyz mares with Brix readings > 20% were classified as having good-quality colostrum (Group 1, *n* = 4), whereas mares with Brix readings < 20% were classified as having inadequate colostrum quality (Group 2, *n* = 4).

The teats of the mares were thoroughly cleaned prior to sampling. Following disinfection with cotton swabs soaked in 70% alcohol, the initial streams of milk were discarded, and approximately 2 mL of milk was aseptically collected into sterile plastic tubes for microbiota analysis. Fresh faecal samples were aseptically collected directly from the rectum of the foals born to these mares. Collected foal faecal and colostrum–milk samples were immediately stored at −80 °C until analysis. During sample collection, each mare and foal initially grazed together in a paddock and subsequently remained in their individual paddocks until completion of the study period.

A total of 79 samples were collected, comprising 40 mare milk samples and 39 foal faecal samples. The sample scheduled for foal F2 at 24 h postpartum (F2_2) was not collected. Milk and faecal samples were collected from eight Kyrgyz mare–foal pairs included in the study. Sampling was performed at five predefined time points to evaluate temporal changes in microbial community composition: immediately after birth (0 h), 24 h postpartum, and days 7, 14, and 21 postpartum. Milk samples obtained from mares were labeled M1–M8, while faecal samples obtained from the corresponding foals were labeled F1–F8. Group 1 contained (M2, M4, M6, M8) and Group 2 contained (M1, M3, M5, M7). Sample identifiers were generated by combining the individual animal code with the sampling time point. For milk samples, the coding format consisted of the mare identification number followed by the sampling time point (e.g., M1_1 indicates the milk sample collected from mare 1 at the first sampling time point, while M8_5 represents the milk sample collected from mare 8 at the fifth sampling time point). The same coding strategy was applied to foal faecal samples, where the prefix “F” denotes faecal samples (e.g., F1_1 indicates the faecal sample obtained from foal 1 at the first sampling time point and F8_5 represents the faecal sample collected from foal 8 at the fifth sampling time point).

### 2.3. DNA Extraction from Colostrum, Milk, and Faecal Samples

Genomic DNA was extracted from colostrum and milk samples using the Milk Bacterial DNA Isolation Kit (Cat. No. 21550; Norgen Biotek, Thorold, ON, Canada) and from faecal samples using the DiaRex Stool Genomic DNA Extraction Kit (Cat. No. SD-0323; Diagen, Ankara, Türkiye), following the respective manufacturers’ instructions. No separate fat-removal step was performed before milk DNA extraction.

### 2.4. Sequencing and Bioinformatic Analysis

The V3–V4 hypervariable region of the bacterial 16S rRNA gene was amplified using primers 341F (5′-CCTAYGGGRBGCASCAG-3′) and 806R (5′-GGACTACNNGGGTATCTAAT-3′) [16]. This region was selected for community-level profiling using paired-end Illumina sequencing. The same primer sequences have also been used to characterise the faecal microbiota of healthy foals [17]. PCR amplification was performed in a total reaction volume of 25 μL containing 12.5 μL KAPA HiFi HotStart Master Mix (Kapa Biosystems; catalogue no. KK2601, Cape Town, South Africa), 0.5 μL of 341F primer (10 μM), 0.5 μL of 806R primer (10 μM), and genomic DNA adjusted to a concentration of 20 ng/µl. The final volume was completed with nuclease-free water. PCR cycling conditions consisted of an initial denaturation at 95 °C for 3 min, followed by 28 cycles of denaturation at 95 °C for 30 s, annealing at 55 °C for 30 s and extension at 72 °C for 45 s, with a final extension step at 72 °C for 10 min. Following amplification, PCR products were purified using AMPure XP magnetic beads (Beckman Coulter) to remove residual primers. Subsequently, Illumina Nextera XT Index Kit v2 Set A (Illumina, Germany) was used to add barcode and index primers to both ends of the PCR products. Indexed amplicons were again purified using AMPure XP beads and diluted to a final concentration of 3.9 ng/μL. Equal volumes (5 μL) of each sample were then pooled to generate the sequencing library. Sequencing was performed on the Illumina MiSeq platform using the MiSeq Reagent Kit v2 (Germany) to obtain 2 × 250 bp paired-end reads. Sequence quality was assessed using FastQC [18]. Reads were processed using QIIME 2 [19], with DADA2 used for quality filtering, denoising, paired-end read merging and chimera removal to infer amplicon sequence variants [20]. Taxonomic assignments were obtained using the SILVA 138 reference database [21,22]. Sample-level read counts at successive processing stages are provided in Supplementary Table S1. Extraction blanks and no-template PCR controls were not included, and no negative-control samples were sequenced. No dedicated bioinformatic procedure was applied to identify and remove potential contaminant ASVs. Consequently, contamination introduced during sampling or laboratory processing could not be directly assessed using negative controls.

### 2.5. Analysis of IgG Concentrations in Milk and Serum

At each microbiota sampling time point, blood samples were aseptically collected from the jugular vein of the foals, and approximately 3 mL of blood was transferred into serum-separator tubes containing a clot activator (BD, Plymouth, UK) for biochemical analysis. Following centrifugation at 3000× *g* for 10 min, the serum was aliquoted and stored at −80 °C until analysis. In addition, following disinfection of the teats with cotton swabs soaked in 70% alcohol, the initial streams of milk were discarded, and an additional approximately 2 mL of milk was also aseptically collected into sterile plastic tubes for the determination of IgG concentrations. IgG concentration in the collected colostrum–milk and foal serum samples was determined using species-specific commercial enzyme-linked immunosorbent assay (ELISA) kit (Cat. no: EA0004Ho, Shanghai YL Biotech Co., Ltd., Shanghai, China) and expressed as mg/mL. The ELISA test procedures were performed in accordance with the manufacturer’s instructions, as outlined in the booklet provided with the commercial kits. After all steps were completed, IgG concentration was measured by reading the plates at 450 nm in an ELISA reader, using this device (Bio-Tek Instruments, Winooski, VT, USA).

IgG concentrations measured in the study were assessed for normality using visual methods (histograms and probability plots) and the Kolmogorov–Smirnov test. As the data did not show a normal distribution, non-parametric statistical tests were applied. Comparisons between Group 1 and Group 2 were performed using the Mann–Whitney U test. For repeated measurements within groups, the Friedman test was used, followed by the Wilcoxon signed-rank test for post hoc pairwise comparisons. Statistical significance was set at *p* < 0.05.

### 2.6. Statistical Analysis

Alpha diversity was assessed using Shannon diversity (log_2_) and observed feature counts at a rarefaction depth of 2000 sequences per sample. Values were averaged across ten rarefaction iterations for each biological sample. Descriptive analyses included 39 milk and 39 faecal samples; M4_3 was excluded because it contained only 686 non-chimeric sequences, whereas F2_2 had not been collected. Temporal differences were evaluated using permutation Friedman tests with 9999 within-animal permutations, including seven mares and seven foals with complete observations at all five time points. Holm correction was applied across the four omnibus tests; no pairwise comparisons were performed.

Beta-diversity inference used weighted UniFrac distance matrices reconstructed from exported pairwise distances. Temporal effects were tested separately for milk and faecal samples using PERMANOVA [23], controlling for animal identity and restricting 9999 permutations within animals. Each analysis included 39 samples from eight animals. Milk–faecal comparisons at each time point included complete mare–foal pairs, controlling for pair identity and enumerating all within-pair label swaps. Effect sizes were calculated as the incremental sum of squares for the tested term divided by the total sum of squares. Multivariate dispersion was assessed using bias-adjusted distances to group spatial medians [24], with residual permutations restricted within animals or pairs, as appropriate. Holm correction was applied separately to temporal and sample-type comparisons for each test.

**Figure 1:**
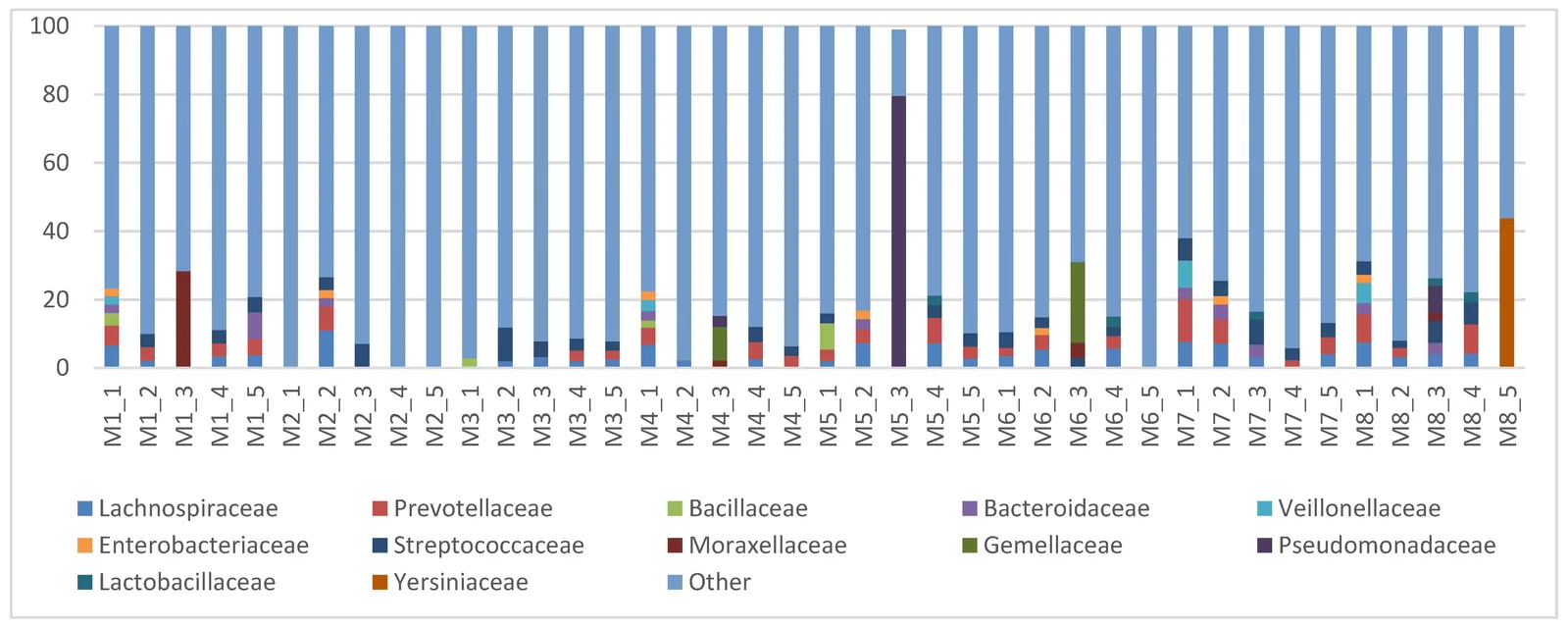
Family-level bacterial DNA profiles detected in 40 mare milk samples at five postpartum time points. Assigned families with a mean relative abundance ≥ 1% in either sample type are displayed individually. “Other” includes lower-abundance assigned taxa, family-level unresolved assignments, and organelle-associated sequences. Percentages are based on total exported sequences without additional filtering. Sample codes M1–M8 identify mares; numbers 1–5 correspond to 0 h, 24 h, and days 7, 14, and 21 postpartum, respectively.

Taxonomic relative abundances were calculated using the complete exported sequence total for each sample, and reported means were unweighted averages across samples. In Figures 1 and 4, assigned family-level taxa with a mean relative abundance of at least 1% in either sample type were displayed individually. The “Other” category combined the remaining lower-abundance assigned taxa, assignments unresolved at the family level, and organelle-associated sequences. Supplementary taxonomic plots display the 15 assigned labels with the highest mean relative abundances across all 79 samples, with *Corynebacteriaceae* additionally retained in the family-level plot. Unlike Figures 1 and 4, Supplementary Figures S2 and S3 display other assigned taxa, taxonomically unresolved assignments, chloroplast-associated sequences, mitochondria-associated sequences, and non-bacterial or domain-unresolved assignments as separate categories. Relative abundances were calculated without additional contaminant filtering.

Statistical analyses were implemented using custom Python 3.0 scripts. Temporal permutation tests assumed exchangeability within animals under the null hypothesis. Observed feature counts could not be independently verified as bacterial ASV richness, and the original rarefaction depth underlying the beta-diversity matrices could not be confirmed. Bray–Curtis ordinations and taxonomic abundance comparisons were descriptive; no differential abundance analysis was performed. Full statistical results and analysis-specific sample sizes are provided in Supplementary Table S2.

## 3. Results

Input read counts ranged from 1209 to 347,530 per milk sample and from 45,998 to 269,567 per faecal sample. Following quality filtering, the corresponding ranges were 1064–283,890 and 39,749–241,391. After chimera removal, 686–163,036 reads per milk sample and 29,058–203,028 reads per faecal sample remained. Sample-level read counts at each processing stage are provided in Supplementary Table S1.

### 3.1. Mare Milk Microbiota

The family-level taxonomic profiles of bacterial DNA detected in mare milk samples are presented in Figure 1. *Lachnospiraceae*, *Prevotellaceae*, and *Streptococcaceae* assignments were detected across the sampling time points in mare M2. *Lachnospiraceae*, *Prevotellaceae*, *Streptococcaceae*, and *Lactobacillaceae* were also among the predominant taxonomic assignments at later sampling time points (Figure 1). In sample M5_3, *Pseudomonadaceae* accounted for 79.6% of the reads in the exported family-level taxonomic table. Several milk samples contained substantial proportions of assignments unresolved at the family level and organelle-associated sequences, which were included in the “Other” category in Figure 1.

At the family level, *Corynebacteriaceae* assignments were detected in all 40 milk samples, with a mean relative abundance of 1.73% and a maximum of 7.32% in M2_3 (Supplementary Figure S2). At the genus level, prominent taxonomic assignments included *Pseudomonas*, *Streptococcus*, *Vibrio*, *Gemella*, and *Corynebacterium*, with mean relative abundances across the 40 milk samples of 4.01%, 3.35%, 2.11%, 2.09%, and 1.72%, respectively. Assignments unresolved at the genus level accounted for a mean of 31.97% of the exported sequences (Supplementary Figure S3).

These taxonomic profiles represent bacterial DNA detected in the analysed milk samples rather than confirmed resident milk-associated microbial communities. Because no sequenced negative controls were available, the potential contribution of background contamination to the observed taxonomic composition and relative abundances could not be determined. Consequently, the biological origin of the detected taxa remains uncertain.

**Figure 2:**
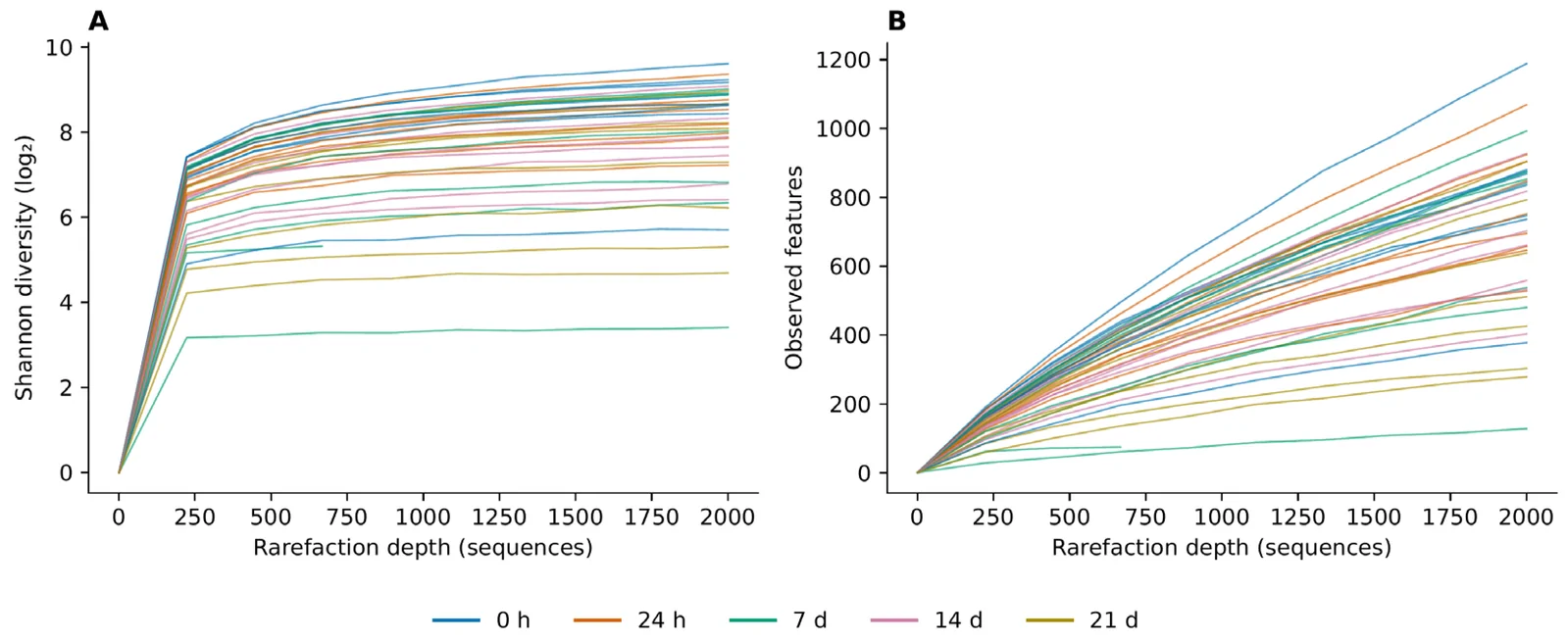
Rarefaction curves of mare milk bacterial DNA profiles showing (**A**) Shannon diversity (log_2_) and (**B**) observed feature counts. Curves represent individual samples averaged over ten rarefaction iterations; colours indicate sampling time. Sample M4_3 (686 non-chimeric sequences) is shown only at depths below its available sequence count. Observed-feature curves did not reach a clear plateau.

No, statistically significant temporal differences were detected in milk-associated bacterial DNA profile diversity, as assessed by Shannon diversity (Friedman Q = 0.914) or observed feature counts (Q = 0.571; Holm-adjusted *p* = 1.000 for both). Observed-feature rarefaction curves continued to increase at the maximum evaluated depth of 2000 sequences per sample without reaching a clear plateau (Figure 2B; Supplementary Figure S1).

**Figure 3:**
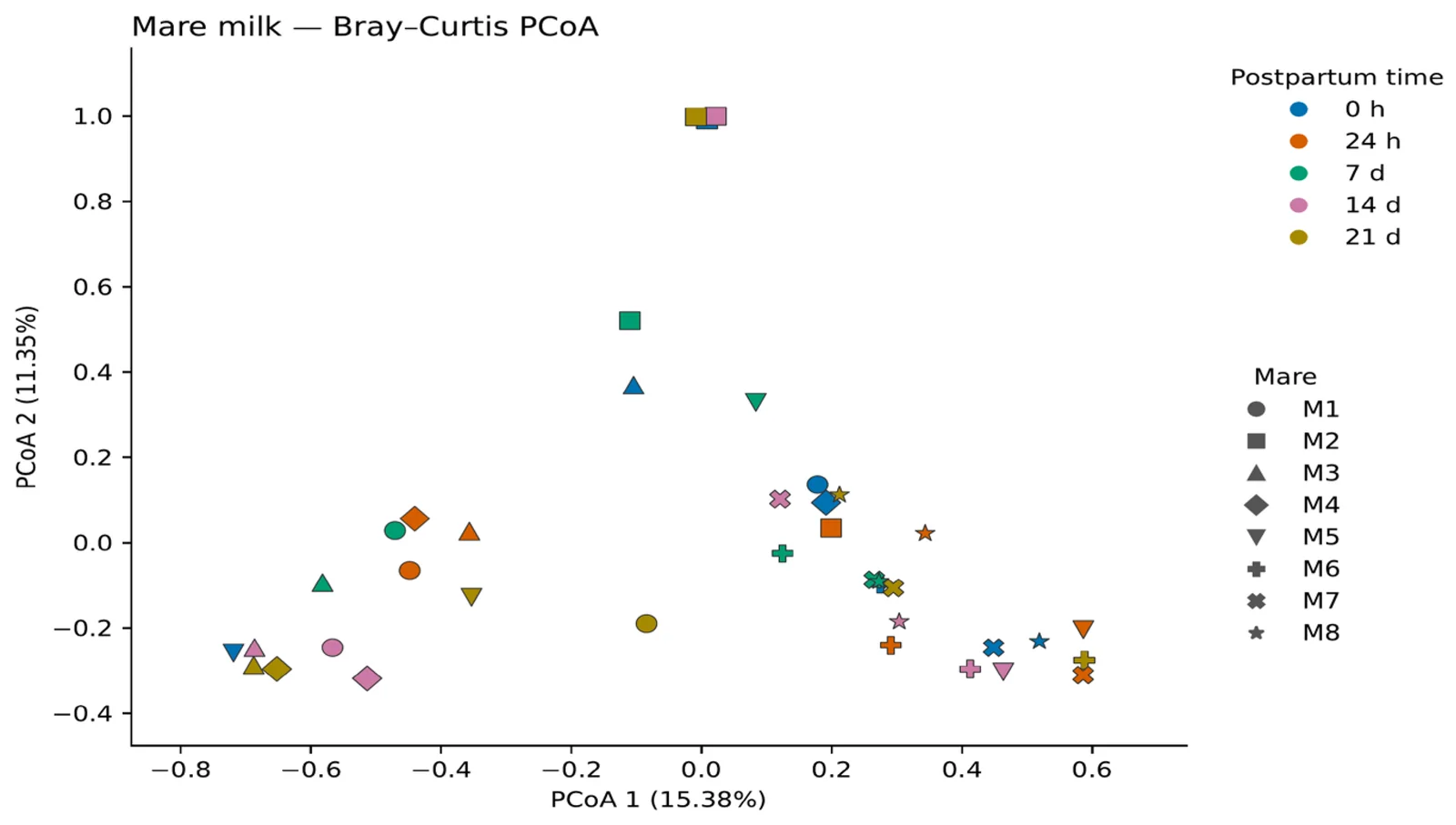
Principal coordinate analysis (PCoA) of mare milk bacterial DNA profiles based on Bray–Curtis dissimilarity. Colours indicate sampling time, and symbols identify individual mares (M1–M8). The first two axes explain 15.38% and 11.35% of the variation, respectively. The analysis includes 39 samples; M4_3 was absent from the supplied ordination output.

Principal coordinate analysis (PCoA) based on Bray–Curtis dissimilarity was used to visualise variation in the bacterial DNA profiles detected in mare milk samples across postpartum sampling times. The first two principal coordinates explained 15.38% and 11.35% of the variation, respectively. Samples collected at different time points overlapped in the ordination, with no clearly separated temporal groups (Figure 3).

Analysis of weighted UniFrac distances did not detect a statistically significant temporal effect on the bacterial DNA profiles detected in milk after controlling for individual identity (PERMANOVA, pseudo-F = 1.318, R^2^ = 0.096, Holm-adjusted *p* = 0.0956). No statistically significant temporal difference in multivariate dispersion was detected (F = 0.493, Holm-adjusted *p* = 0.4663). These statistical findings describe variation in the observed sequencing profiles and should not be interpreted as evidence of temporal stability in the resident milk microbiota.

### 3.2. Foal Faecal Microbiota

**Figure 4:**
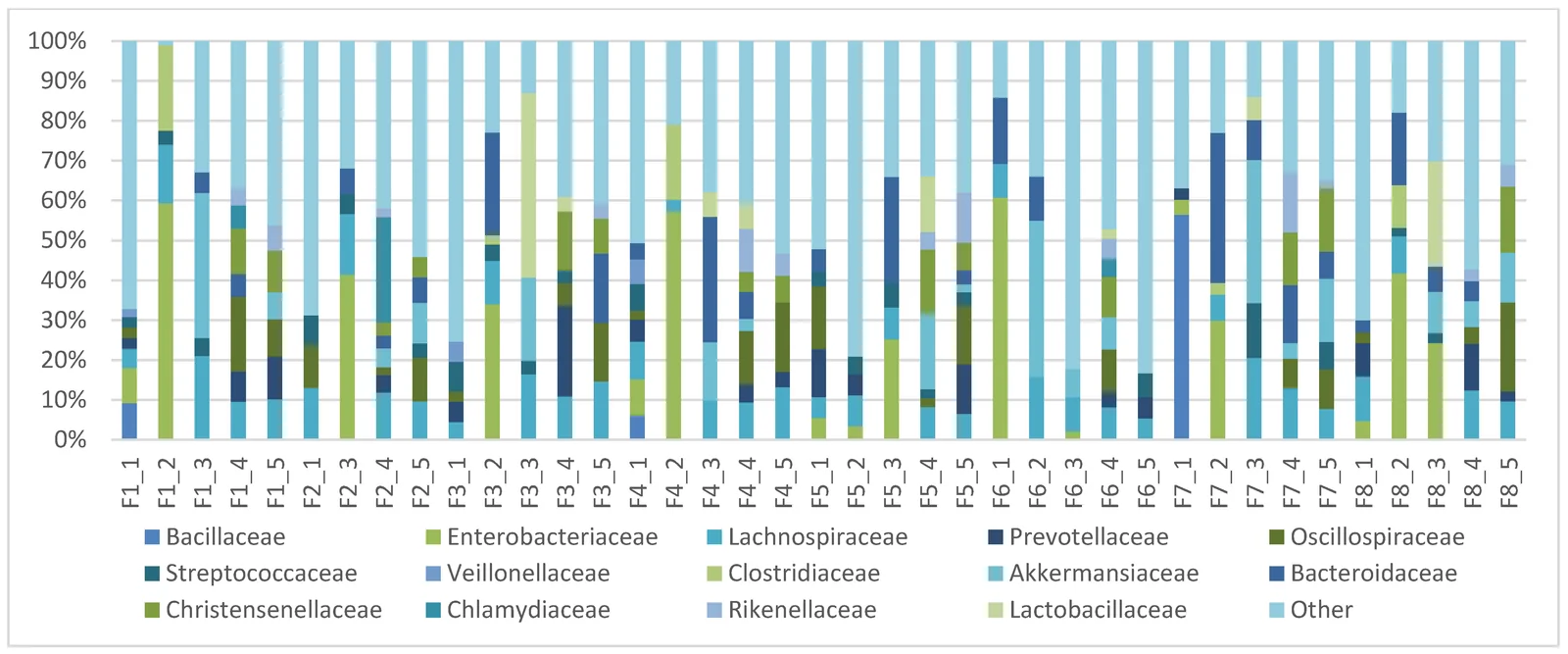
Family-level bacterial DNA profiles detected in 39 foal faecal samples at five postpartum time points. Assigned families with a mean relative abundance ≥ 1% in either sample type are displayed individually. “Other” includes lower-abundance assigned taxa, family-level unresolved assignments, and organelle-associated sequences. Percentages are based on total exported sequences without additional filtering. Sample codes F1–F8 identify foals; numbers 1–5 correspond to 0 h, 24 h, and days 7, 14, and 21 postpartum, respectively. F2_2 was not collected; the gap does not represent zero abundance.

Foal faecal bacterial community profiles varied across the five sampling time points during the first 21 days postpartum. *Enterobacteriaceae*, *Bacillaceae*, and *Streptococcaceae* were among the prominent bacterial families during the early sampling period (Figure 4). *Enterobacteriaceae* exceeded 60% relative abundance in F1_2 (62.2%) and F6_1 (61.8%). At 24 h postpartum, *Clostridiaceae* reached 22.3% in F1_2 and 19.7% in F4_2. At the same sampling time, other foals showed appreciable proportions of *Lachnospiraceae*, *Bacteroidaceae and Akkermansiaceae*. At day 7, *Lactobacillaceae*, *Akkermansiaceae*, and *Bacteroidaceae* reached relative abundances of up to 46.6%, 36.7% and 31.3%, respectively, in individual samples. At days 14 and 21, *Lachnospiraceae*, *Oscillospiraceae*, *Christensenellaceae*, *Rikenellaceae*, *Prevotellaceae* and *Akkermansiaceae* were among the prominent bacterial families (Figure 4). At genus level, prominent assigned taxa included *Escherichia–Shigella*, *Akkermansia* and *Bacteroides*, with mean relative abundances across the 39 faecal samples of 10.33%, 7.24% and 7.08%, respectively. Assignments unresolved at genus level accounted for a mean of 17.13% of the exported sequences (Supplementary Figure S3).

**Figure 5:**
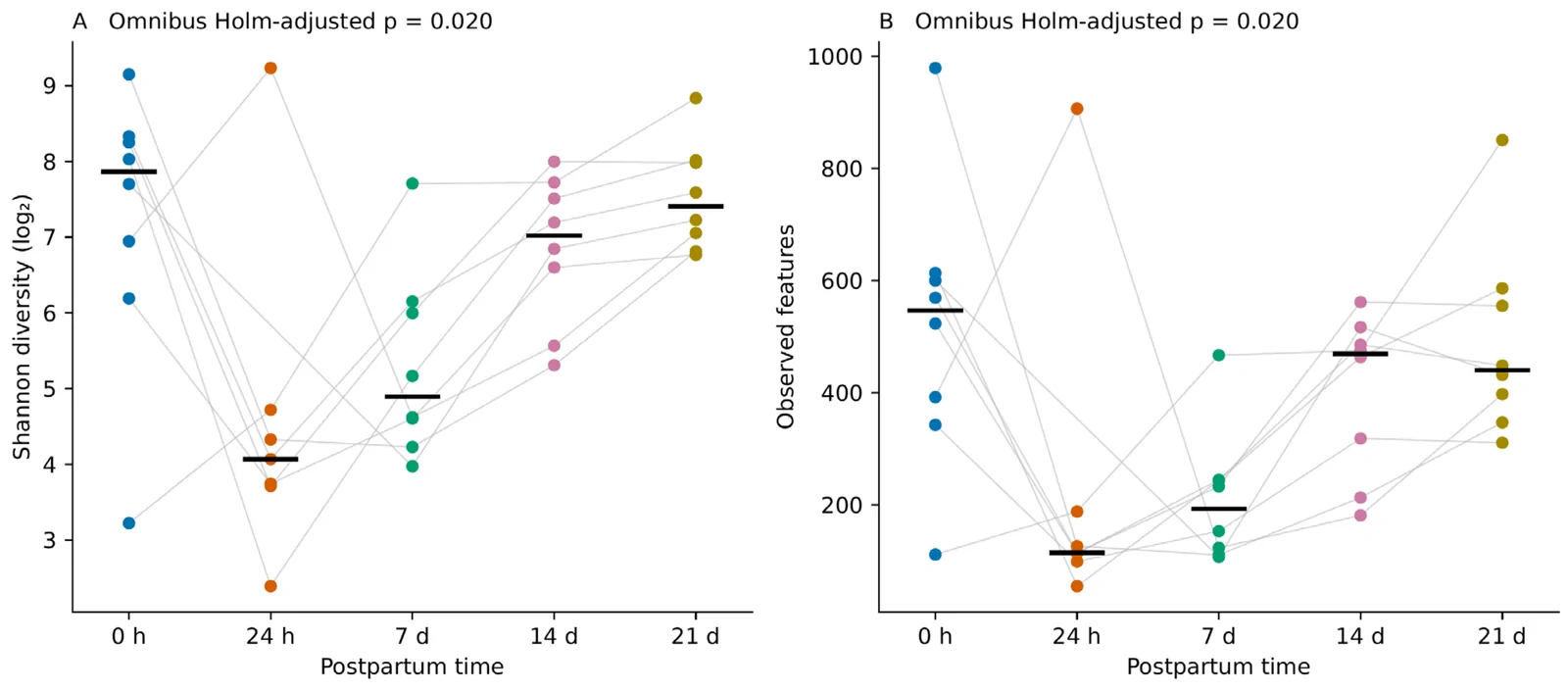
Foal faecal alpha diversity during the first 21 days postpartum: (**A**) Shannon diversity (log_2_) and (**B**) observed feature counts. Points represent sample-level means across ten rarefaction iterations at 2000 sequences per sample. Colours indicate sampling time, grey lines connect observations from the same foal, and black horizontal bars indicate time-point medians. All 39 available samples are shown: eight per time point, except at 24 h (*n* = 7). Omnibus p-values were obtained using permutation Friedman tests restricted to the seven foals with complete observations, with Holm adjustment across the four milk and faecal alpha-diversity tests. No pairwise comparisons were performed.

In complete-case permutation analyses, temporal differences were detected in foal faecal Shannon diversity (Friedman Q = 13.257, Holm-adjusted *p* = 0.020) and observed feature counts (Q = 12.914, Holm-adjusted *p* = 0.020). Median values were descriptively lower at 24 h and 7 d than at birth and increased again at 14–21 d (Figure 5). These omnibus tests indicate an overall temporal difference but do not identify which individual time points differed.

**Figure 6:**
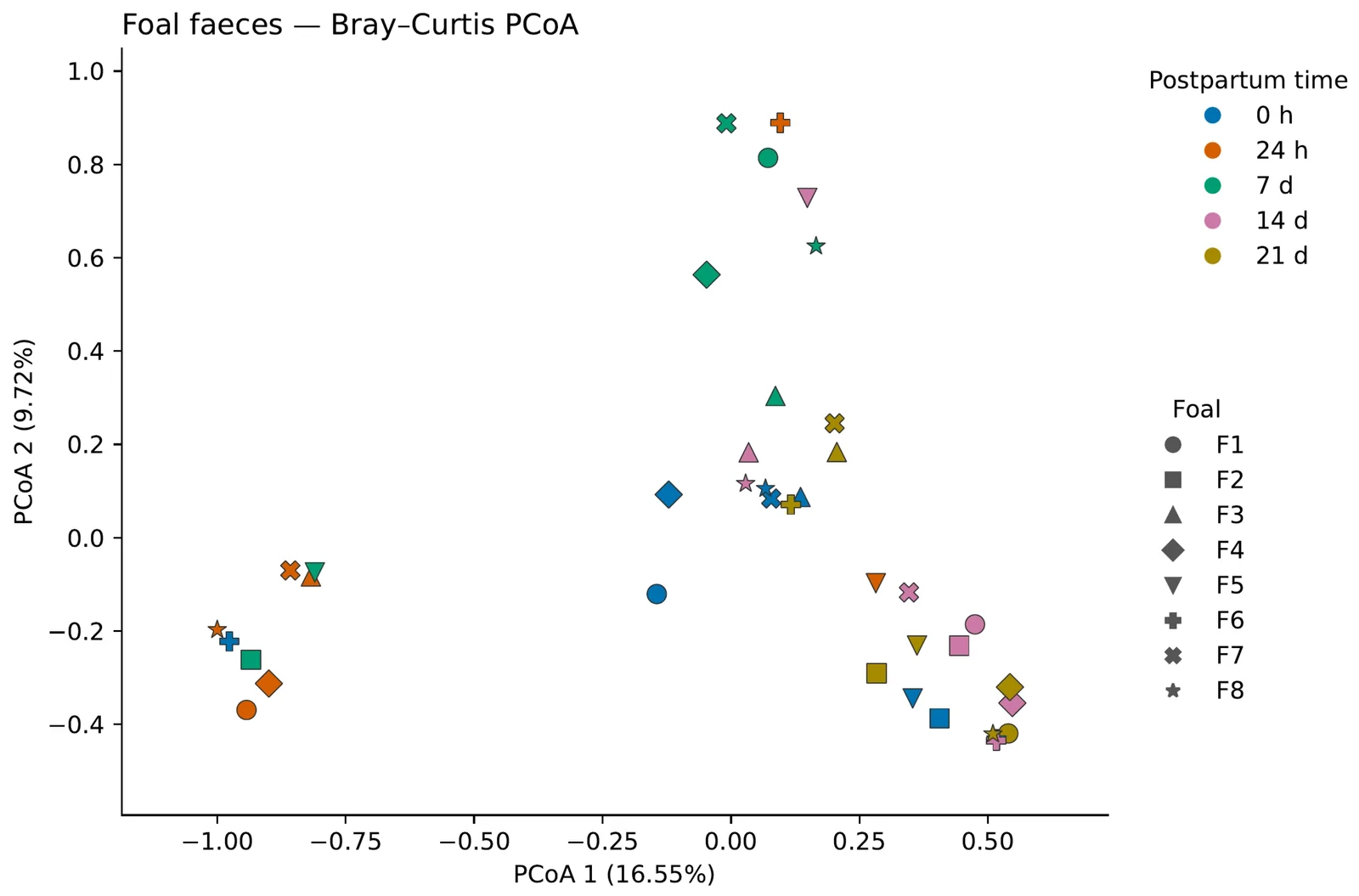
Principal coordinate analysis (PCoA) of foal faecal bacterial community profiles based on Bray–Curtis dissimilarity. Each point represents an individual faecal sample. Colours indicate postpartum sampling time, and symbols identify individual foals (F1–F8), as shown in the legends. The first two principal coordinates explain 16.55% and 9.72% of the variation, respectively. The plot includes 39 samples; F2_2 was not collected.

Principal coordinate analysis (PCoA) based on Bray–Curtis dissimilarity was used to visualise variation in foal faecal bacterial community composition across sampling times. The first two principal coordinates explained 16.55% and 9.72% of the variation, respectively (Figure 6).

Analysis of weighted UniFrac distances detected a temporal effect on foal faecal community composition after controlling for individual identity (PERMANOVA, pseudo-F = 2.271, R^2^ = 0.218, Holm-adjusted *p* = 0.0028). No statistically significant temporal difference in multivariate dispersion was detected (F = 1.924, Holm-adjusted *p* = 0.1428).

### 3.3. Maternal Milk–Foal Faecal Microbiota

**Figure 7:**
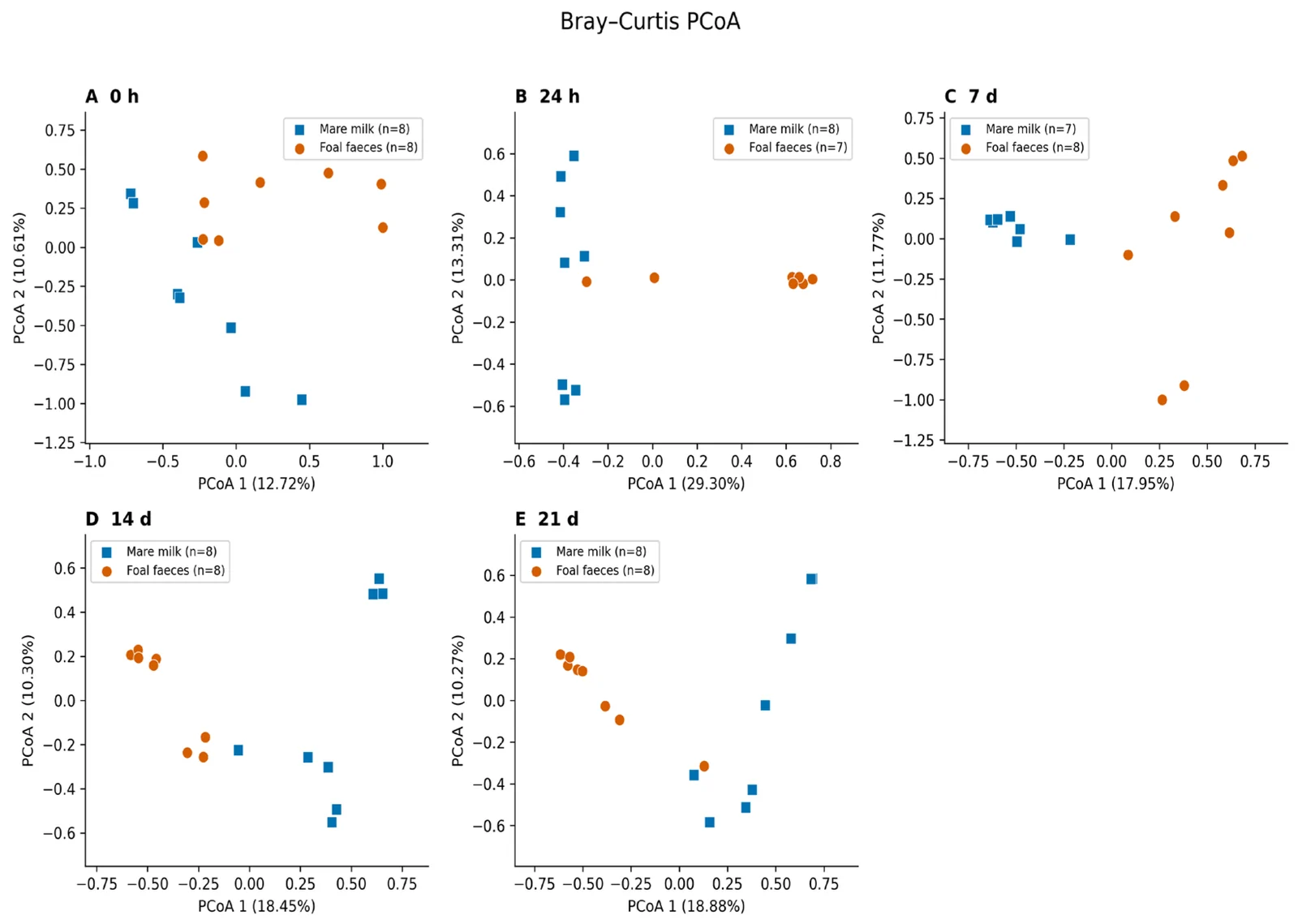
Principal coordinate analysis (PCoA) of bacterial DNA profiles detected in mare milk and foal faecal samples, based on Bray–Curtis dissimilarity at (**A**) 0 h, (**B**) 24 h, (**C**) 7 d, (**D**) 14 d, and (**E**) 21 d postpartum. Each point represents one sample; blue squares indicate mare milk and orange circles indicate foal faeces. Sample counts are shown in each panel. All available samples are displayed, whereas statistical comparisons included only complete mare–foal pairs. Ordinations were calculated separately for each time point, with the explained variation indicated on each axis. F2_2 was not collected, and M4_3 was absent from the supplied ordination output. These descriptive plots do not test temporal convergence.

Bray–Curtis principal coordinate analysis was used to visualise differences between bacterial DNA profiles detected in mare milk and foal faecal samples at each sampling time (Figure 7). Pair-blocked PERMANOVA based on weighted UniFrac distances detected differences between sample types at all five sampling times (Holm-adjusted *p* = 0.0391 for each comparison). However, multivariate dispersion also differed between sample types at every time point (Holm-adjusted *p* = 0.0195–0.0313), with greater dispersion in milk. Consequently, the significant PERMANOVA results cannot be attributed exclusively to differences in multivariate location. Full test results, effect sizes, and analysis-specific sample sizes are provided in Supplementary Table S2.

Taxonomic overlap was observed between the bacterial DNA profiles detected in mare milk and foal faecal samples. *Firmicutes* and *Bacteroidota* were identified in both sample types, while shared family-level assignments included *Lachnospiraceae*, *Streptococcaceae*, and *Prevotellaceae* at multiple sampling times. However, because sequenced negative controls were unavailable, the contribution of potential background contamination to the milk profiles could not be determined. Therefore, these similarities represent descriptive overlap between detected bacterial DNA sequences and cannot establish microbial transmission, milk-derived intestinal colonisation, or a direct biological relationship between the two sample types. Neither the separate time-point comparisons nor the ordinations demonstrate temporal convergence.

Expanded family- and genus-level taxonomic profiles are presented in Supplementary Figures S2 and S3, respectively. These displays distinguish other assigned taxa from unresolved assignments, organelle-associated sequences, and non-bacterial or domain-unresolved assignments. Relative abundances were calculated using the complete exported sequence total for each sample; these categories were separated for transparency rather than removed through additional filtering.

### 3.4. Foal Serum and Mare Milk Immunoglobulin G Concentrations

Changes in IgG concentrations in mares and foals from Groups 1 and 2, based on intergroup comparisons and postpartum time points, are presented in Supplementary Figures S4 and S5, respectively.

Higher colostral IgG concentrations were detected in Group 1, in which Brix values exceeded 20%. However, in both groups, colostral IgG concentrations declined rapidly after 24h postpartum and remained at low levels thereafter in both groups (Supplementary Figure S4). In both groups of mares, a rapid decline in milk IgG concentrations was observed after 24h postpartum.

In foals, ingestion of colostrum with a high IgG concentration resulted in significantly higher serum IgG levels at 24h of age. This difference between groups remained evident throughout the postpartum period. Postpartum changes in serum IgG concentrations in foals are shown in Supplementary Figure S5. In both groups, serum IgG concentrations were very low at 0 h postpartum. From 24 h onward, serum IgG levels increased markedly and remained elevated throughout the 21-day period. However, foals that received colostrum with lower IgG concentrations (Group 2) consistently exhibited lower serum IgG levels compared with Group 1 foals throughout the entire 21-day postpartum period.

## 4. Discussion

This pilot study characterised bacterial DNA profiles detected in mare milk and foal faecal samples during the first 21 days postpartum. Statistically significant temporal differences were detected in foal faecal alpha diversity and weighted UniFrac profiles, whereas the corresponding tests in milk did not reach statistical significance. However, these findings were obtained from separate analyses, and no direct sample type-by-time interaction was tested. Therefore, they do not demonstrate that the temporal responses differed significantly between milk and faecal samples. Furthermore, the absence of statistical significance in milk should not be interpreted as evidence of temporal stability, particularly given the small sample size and uncertainty surrounding the biological origin of milk-associated bacterial DNA. Milk–faecal comparisons were significant at each sampling time, although differences in multivariate dispersion limit their interpretation. Accordingly, these results should be regarded as exploratory.

Mienaltowski et al. [14] provide a particularly relevant comparison because they examined paired mare milk and foal rectal-swab samples longitudinally. Their milk profiles were dominated by *Escherichia–Shigella*, and their Figure 2 shows milk Chao1 central values generally below or around 100 and Shannon values around 3.5–4. Milk and foal communities were initially similar but subsequently diverged, leading the authors to emphasise sources outside milk in the developing foal gut microbiota. In contrast, our milk profiles included prominent gut-associated families such as *Lachnospiraceae*, *Prevotellaceae*, and *Bacteroidaceae*. These differences cannot be attributed solely to biological variation between populations. Sampling procedures, extraction methods, target regions and bioinformatic processing may contribute, while the absence of negative controls leaves contamination unresolved. Direct numerical comparisons of diversity also require caution: our revised richness measure is observed features rather than Chao1, and our Shannon values use log_2_, requiring harmonisation of logarithm bases before comparison. The present data therefore do not establish greater biological richness in milk or a colonisation trajectory opposing that proposed by Mienaltowski et al. [14].

The lack of statistically significant temporal effects in milk does not establish stability, equivalence or ecological resilience. With only eight mares, and seven contributing complete series to the alpha-diversity tests, modest changes may have remained undetected. Similarly, repeated detection of *Firmicutes*, *Bacteroidota*, *Lachnospiraceae*, *Prevotellaceae*, and *Streptococcaceae* does not establish a conserved resident milk microbiota. Family-level assignments encompass organisms with different ecological and clinical characteristics; their presence alone cannot distinguish commensal populations, environmental introduction or potentially pathogenic organisms.

The dominance of Pseudomonadaceae in M5_3, accounting for 79.6% of reads in the exported family-level table, requires particular caution. Li et al. [25] reported abundant Pseudomonas in water deer milk, showing that this genus has been detected in milk from another mammalian species. However, this observation does not establish the origin of Pseudomonadaceae in our sample. Salter et al. [26] identified Pseudomonas among reagent-associated contaminants and demonstrated the vulnerability of low-biomass profiles to background DNA. Scully et al. [27] included negative controls and computational contaminant removal when characterising processed bovine colostrum, whereas these measures were not incorporated into the present study. Without extraction blanks, no-template PCR controls or corresponding environmental samples, the M5_3 profile cannot distinguish biological variation from contamination during collection or processing. Its high relative abundance also does not demonstrate an increased absolute bacterial load.

The diversity and taxonomic summaries have additional analytical constraints. Milk observed-feature rarefaction curves continued to increase at 2000 sequences per sample, so this depth did not provide evidence of complete richness sampling. Standardising sequencing depth supports comparisons within the analysed dataset but does not establish that all taxa were adequately represented. In the main family-level plots (Figure 1 and Figure 4), “Other” combines lower-abundance assigned taxa, sequences unresolved at the family level, and organelle-associated sequences. The expanded supplementary profiles separate remaining assigned taxa from unresolved, organelle-associated and non-bacterial or domain-unresolved assignments. The size of the “Other” category in the main plots therefore reflects both the grouping rule and taxonomic resolution and cannot independently demonstrate ecological complexity, sequencing adequacy or poor sample quality. Interpretation of uncommon taxa should remain descriptive until their assignments and potential technical origins can be examined.

Foal faecal profiles showed temporal changes consistent with the broader evidence that early equine gut microbial composition varies with age [12,13]. *Enterobacteriaceae*, *Bacillaceae*, and *Streptococcaceae* predominated during the early sampling period, whereas *Lachnospiraceae*, *Oscillospiraceae*, *Christensenellaceae*, *Rikenellaceae*, *Prevotellaceae*, and *Bacteroidaceae* became more prominent in several later samples. De La Torre et al. [12] similarly described a transition from early *Proteobacteria*-dominated profiles towards communities containing more *Firmicutes* during development. These broad similarities support comparison with neonatal microbial succession, but they do not establish identical trajectories across studies. Family-level relative abundance profiles do not establish metabolic activity or changes in intestinal oxygen availability. Because differential abundance analysis was not performed, the observed taxonomic shifts should be interpreted descriptively rather than as statistically demonstrated enrichment of individual families.

Importantly, foal alpha diversity did not increase progressively from birth. Median Shannon diversity and observed feature counts were higher at 0 h than at 24 h and 7 d and increased again at 14–21 d. The omnibus tests support overall temporal differences but do not identify particular time-point contrasts. The relatively high diversity values at 0 h warrant caution because bacterial biomass was not quantified, leaving the susceptibility of these earliest samples to background DNA unresolved. Background DNA can disproportionately influence low-biomass profiles [26]; consequently, the 0 h results cannot be assumed to represent a highly diverse, established intestinal community or evidence of prenatal colonisation. Biological and technical contributions to this pattern remain unresolved.

Milk and faecal profiles shared bacterial families, including *Lachnospiraceae*, *Streptococcaceae*, and *Prevotellaceae*, but this overlap does not identify their source or direction of transfer. Neither the separate time-point comparisons nor the independently calculated ordinations establish temporal convergence between paired milk and faecal profiles. Changes within foal faecal profiles must therefore be distinguished from changes in milk–faecal similarity. Mady et al. [28] examined associations between mare milk metabolites and foal gut microbiota, addressing a potential nutritional relationship rather than demonstrating bacterial transmission through milk. In humans, Ferretti et al. [29] used longitudinal, strain-resolved metagenomics across maternal and infant body sites to investigate microbial transmission. Although those findings cannot be directly extrapolated to horses, they illustrate the additional resolution and source sampling required beyond shared family-level assignments.

Maternal faecal samples represent another potential source of microbes for the developing foal gut. Pyles et al. [30] detected coprophagy from day 3 postpartum and found a positive association between maternal faecal consumption and culturable cellulolytic bacteria in foal faecal samples at day 7. These findings support a potential role for coprophagy in early microbial establishment, although they do not identify the origin of taxa detected in the present study. Because maternal faecal microbiota was not sampled, its potential contribution could not be evaluated or distinguished from milk-associated and other environmental sources.

Several limitations constrain interpretation. The small cohort and 21-day follow-up limit generalisability and assessment of later persistence. Primer selection can influence the taxonomic coverage of 16S rRNA gene surveys [31]. Partial 16S rRNA gene sequences may provide insufficient resolution to distinguish closely related species or strains [32]. Accordingly, ASVs should not be interpreted as direct equivalents of species or strains. Relative sequence abundance does not quantify bacterial load, and DNA detection does not establish viability. The absence of sequenced extraction blanks and no-template PCR controls is a major limitation, particularly for milk and potentially low-biomass samples collected at birth. Without these controls or bacterial biomass measurements, the contribution of background DNA to the observed profiles could not be reliably assessed [26]. The milk findings therefore remain exploratory, with unresolved biological and technical contributions. The lack of maternal faecal, teat-skin and environmental samples further restricts source attribution. Statistical reanalysis relied on exported diversity values and distance matrices, preventing independent verification of bacterial feature filtering and the original beta-diversity rarefaction depth. Complete-case alpha-diversity tests reduced the sample size, and restricted permutations assumed within-animal exchangeability without explicitly modelling serial autocorrelation. Future studies incorporating larger cohorts, longer follow-up, bacterial-load measurements, sequenced negative controls and strain-resolved source comparisons are needed to investigate whether milk-associated bacteria contribute to foal gut colonisation. Another one limitation of this study is that the nutritional composition of the individual feed ingredients and the complete ration was not determined by laboratory analysis. Although all mares were maintained at the same breeding facility and received the same feeding regimen, the absence of analytical data on crude protein, crude fiber, ash, NDF, ADF, and other nutritional parameters precluded a detailed assessment of dietary composition. Therefore, potential effects of unmeasured nutritional and animal-related factors cannot be completely excluded when interpreting the findings.

## 5. Conclusions

This exploratory pilot study characterised bacterial DNA profiles in mare milk and foal faecal samples from eight Kyrgyz mare–foal pairs during the first 21 days postpartum. Significant temporal differences were detected in foal faecal diversity and weighted UniFrac profiles, whereas corresponding analyses of milk profiles did not reach statistical significance. However, these separate findings do not establish a difference in temporal responses between sample types, as no direct interaction test was performed. Milk–faecal comparisons also yielded significant results, although differences in multivariate dispersion limit their interpretation. Shared taxonomic assignments do not demonstrate microbial transmission or milk-derived intestinal colonisation. Furthermore, the absence of sequenced negative controls prevents reliable differentiation between genuine milk-associated bacterial DNA and potential background contamination. Given these limitations and the small cohort, the findings should be interpreted as exploratory observations requiring validation in larger, appropriately controlled longitudinal studies.

## Data Availability

All raw 16S rRNA gene sequencing data generated in the present study have been deposited in the National Center for Biotechnology Information (NCBI) Sequence Read Archive (SRA). The sequencing data are publicly accessible under BioProject accession number PRJNA1498830, with corresponding BioSample accession numbers SAMN61859517–SAMN61859595 and SRA accession numbers SRR39758556–SRR39758634.

## Supplementary Materials

The following supporting information can be downloaded at https://www.mdpi.com/article/10.3390/ani16213448/s1.

## Abbreviations

PCoA	Principal coordinate analysis
ASVs	Amplicon sequence variants
BRM	Brix refractometer
IgG	Immunoglobulin G

## References

[1] Sievert M., Krohn J., Wehrend A. (2019). Immunglobulinkonzentration Im Equinen Kolostrum Und Im Blut Neugeborener Fohlen Sowie Klinisch Relevante IgG-Bestimmungsmethoden. Tierärztl. Prax. Ausg. G Großtiere/Nutztiere.

[2] Chucri T.M., Monteiro J.M., Lima A.R., Salvadori M.L., Kfoury J.R., Miglino M.A. (2010). A Review of Immune Transfer by the Placenta. J. Reprod. Immunol..

[3] McClure J.T., Sellon D.C., Hines M.T., Smith B.P. (2002). Immunologic Disorders, Equine Immunodeficiency Diseases. Large Animal Internal Medicine.

[4] Kelly G.S. (2003). Bovine Colostrum: A Review of Clinical Uses. Altern. Med. Rev..

[5] Giguère S., Polkes A.C. (2005). Immunologic Disorders in Neonatal Foals. Vet. Clin. North Am. Equine Pract..

[6] Gamsjäger L., Elsohaby I., Pearson J.M., Levy M., Pajor E.A., Windeyer M.C. (2021). Evaluation of 3 Refractometers to Determine Transfer of Passive Immunity in Neonatal Beef Calves. J. Vet. Intern. Med..

[7] Turini L., Nocera I., Bonelli F., Mele M., Sgorbini M. (2020). Evaluation of Brix Refractometry for the Estimation of Colostrum Quality in Jennies. J. Equine Vet. Sci..

[8] Cash R.S.G. (1999). Colostral Quality Determined by Refractometry. Equine Vet. Educ..

[9] Perkins G.A., Wagner B. (2015). The Development of Equine Immunity: Current Knowledge on Immunology in the Young Horse. Equine Vet. J..

[10] Paradis M.R., Sellon D.C. (2006). Neonatal Immunology. Equine Neonatal Medicine: A Case-Based Approach.

[11] Davis R., Giguère S. (2005). Evaluation of Five Commercially Available Assays and Measurement of Serum Total Protein Concentration via Refractometry for the Diagnosis of Failure of Passive Transfer of Immunity in Foals. J. Am. Vet. Med. Assoc..

[12] De La Torre U., Henderson J.D., Furtado K.L., Pedroja M., Elenamarie O.M., Mora A., Pechanec M.Y., Maga E.A., Mienaltowski M.J. (2019). Utilizing the Fecal Microbiota to Understand Foal Gut Transitions from Birth to Weaning. PLoS ONE.

[13] O’Reilly G.C., Holman D.B., Muscat K., Muscatello G., Parra M.C., Meale S.J., Chaves A.V. (2022). Characterisation of the Faecal Microbiome of Foals from 0–5 Months of Age and Their Respective Mares across Five Geographic Locations. Front. Biosci..

[14] Mienaltowski M.J., Callahan M., De La Torre U., Maga E.A. (2024). Comparing Microbiotas of Foals and Their Mares’ Milk in the First Two Weeks after Birth. BMC Vet. Res..

[15] Yüksel M., Saat N., Kaymaz M., Fındık M., Rişvanlı A., Köker A. (2017). Beslenmenin Fertilite Üzerine Etkisi ve Haralarda Reprodüksiyon Yönetimi. Kısraklarda Doğum ve Jinekoloji.

[16] Xu Y., Moser C., Al-Soud W.A., Sørensen S., Høiby N., Nielsen P.H., Thomsen T.R. (2012). Culture-Dependent and -Independent Investigations of Microbial Diversity on Urinary Catheters. J. Clin. Microbiol..

[17] Liu Y., Bailey K.E., Dyall-Smith M., Marenda M.S., Hardefeldt L.Y., Browning G.F., Gilkerson J.R., Billman-Jacobe H. (2021). Faecal microbiota and antimicrobial resistance gene profiles of healthy foals. Equine Vet. J..

[18] Andrews S. (2010). FastQC: A Quality Control Tool for High Throughput Sequence Data. https://www.bioinformatics.babraham.ac.uk/projects/fastqc/.

[19] Bolyen E., Rideout J.R., Dillon M.R., Bokulich N.A., Abnet C.C., Al-Ghalith G.A., Caporaso J.G. (2019). Reproducible, Interactive, Scalable and Extensible Microbiome Data Science Using QIIME 2. Nat. Biotechnol..

[20] Callahan B.J., McMurdie P.J., Rosen M.J., Han A.W., Johnson A.J.A., Holmes S.P. (2016). DADA2: High-Resolution Sample Inference from Illumina Amplicon Data. Nat. Methods.

[21] Chuvochina M., Gerken J., Frentrup M., Sandikci Y., Goldmann R., Freese H.M., Göker M., Sikorski J., Yarza P., Quast C. (2026). SILVA in 2026: A global core biodata resource for rRNA within the DSMZ digital diversity. Nucleic Acids Res..

[22] Quast C., Pruesse E., Yilmaz P., Gerken J., Schweer T., Yarza P., Glöckner F.O. (2012). The SILVA Ribosomal RNA Gene Database Project: Improved Data Processing and Web-Based Tools. Nucleic Acids Res..

[23] Anderson M.J. (2001). A new method for non-parametric multivariate analysis of variance. Austral Ecol..

[24] Anderson M.J. (2006). Distance-based tests for homogeneity of multivariate dispersions. Biometrics.

[25] Li Z., Wright A.-D.G., Yang Y., Si H., Li G. (2017). Unique Bacteria Community Composition and Co-Occurrence in the Milk of Different Ruminants. Sci. Rep..

[26] Salter S.J., Cox M.J., Turek E.M., Calus S.T., Cookson W.O., Moffatt M.F., Turner P., Parkhill J., Loman N.J., Walker A.W. (2014). Reagent and laboratory contamination can critically impact sequence-based microbiome analyses. BMC Biol..

[27] Scully S., Earley B., Smith P.E., Finnie M.S., McAloon C., Kenny D.A., Waters S.M. (2026). Characterisation of the bacterial and archaeal microbiota in processed colostrum collected from a spring-calving dairy herd. PLoS ONE.

[28] Mady E.A., Osuga H., Toyama H., El-Husseiny H.M., Inoue R., Murase H., Nagaoka K. (2024). Relationship between the Components of Mare Breast Milk and Foal Gut Microbiome: Shaping Gut Microbiome Development after Birth. Vet. Q..

[29] Ferretti P., Pasolli E., Tett A., Asnicar F., Gorfer V., Fedi S., Armanini F., Truong D.T., Manara S., Zolfo M. (2018). Mother-to-infant microbial transmission from different body sites shapes the developing infant gut microbiome. Cell Host Microbe.

[30] Pyles M., Agbana M., Hayes S., Flythe M., Lawrence L. (2023). The Establishment of Fibrolytic Bacteria in the Foal Gastrointestinal Tract Is Related to the Occurrence of Coprophagy by Foals. Animals.

[31] Klindworth A., Pruesse E., Schweer T., Peplies J., Quast C., Horn M., Glöckner F.O. (2013). Evaluation of general 16S ribosomal RNA gene PCR primers for classical and next-generation sequencing-based diversity studies. Nucleic Acids Res..

[32] Johnson J.S., Spakowicz D.J., Hong B.-Y., Petersen L.M., Demkowicz P., Chen L., Leopold S.R., Hanson B.M., Agresta H.O., Gerstein M. (2019). Evaluation of 16S rRNA gene sequencing for species and strain-level microbiome analysis. Nat. Commun..

