# Supplementary material for: Comparative Analysis of Mare Milk and Foal Faecal Microbiota in Kyrgyz Mare–Foal Pairs During the First 21 Days Postpartum
Source: Animals (Basel). 2026 Nov 1;16(21):3448. doi: 10.3390/ani16213448 (PMC13761987; doi:10.3390/ani16213448)
Supplement: Supplementary file 1 [file animals-16-03448-s001.zip › Supplementary Figure S1.pdf]

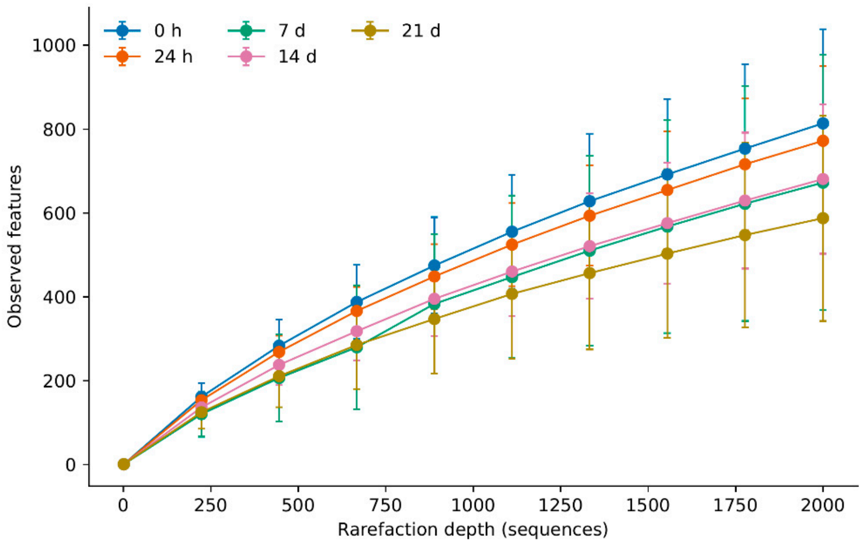

Supplementary Figure S1. Observed-feature rarefaction curves for mare milk samples grouped by postpartum sampling time. Within each sample, values were averaged across ten rarefaction iterations at each sequencing depth. Points and error bars represent the mean and standard deviation among available samples. Eight samples contribute at each time point, except at 7 d at evaluated depths above 686 sequences, where seven samples contribute because M4.3 is unavailable. These curves describe observed feature recovery and do not establish the total bacterial richness of the samples.
