# Supplementary material for: Comparative Analysis of Mare Milk and Foal Faecal Microbiota in Kyrgyz Mare–Foal Pairs During the First 21 Days Postpartum
Source: Animals (Basel). 2026 Nov 1;16(21):3448. doi: 10.3390/ani16213448 (PMC13761987; doi:10.3390/ani16213448)
Supplement: Supplementary file 1 [file animals-16-03448-s001.zip › Supplementary Figure S2.pdf]

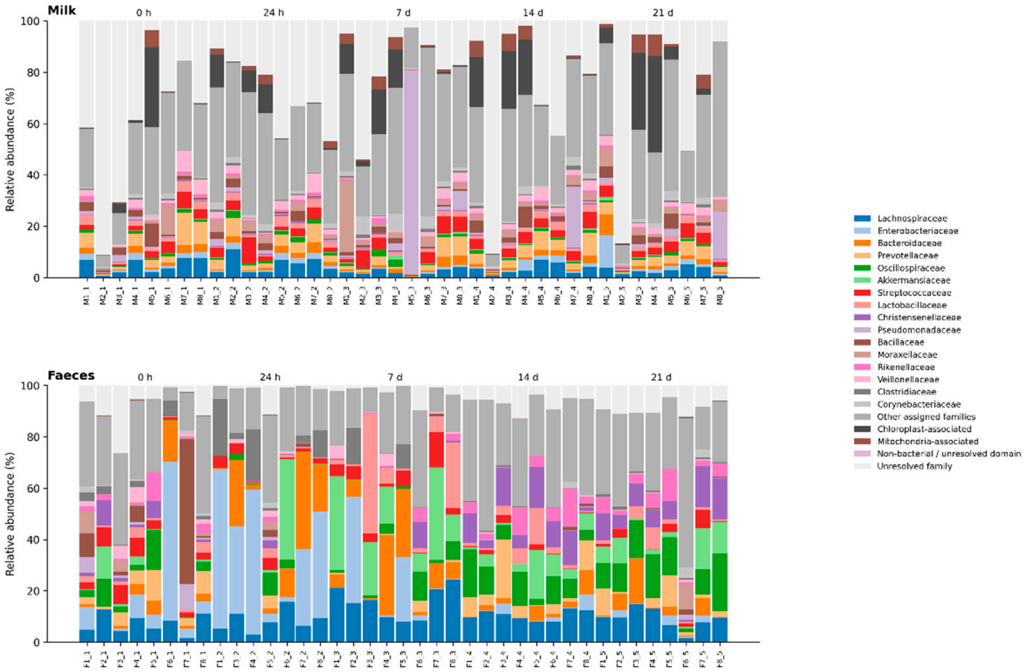

Supplementary Figure S2: Family-level composition of mare milk and foal faecal profiles. Each bar represents one sample, with relative abundances calculated using its complete exported sequence total. The 15 assigned family labels with the highest mean relative abundances across all 79 samples are displayed individually, together with Corynebacteriaceae. Remaining assigned families are grouped as “Other assigned families”. Unresolved family assignments, chloroplast-associated sequences, mitochondria-associated sequences and non-bacterial or domain-unresolved assignments are shown separately. Sampling numbers 1–5 correspond to 0 h, 24 h, 7 d, 14 d and 21 d postpartum. All 40 milk and 39 faecal samples are included.
