# Supplementary material for: Comparative Analysis of Mare Milk and Foal Faecal Microbiota in Kyrgyz Mare–Foal Pairs During the First 21 Days Postpartum
Source: Animals (Basel). 2026 Nov 1;16(21):3448. doi: 10.3390/ani16213448 (PMC13761987; doi:10.3390/ani16213448)
Supplement: Supplementary file 1 [file animals-16-03448-s001.zip › Supplementary Figure S4.pdf]

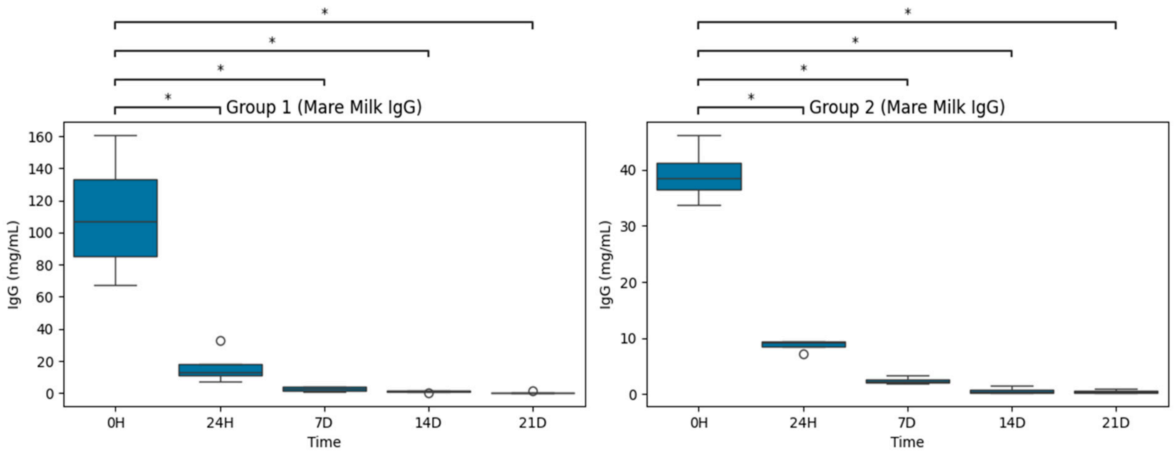

Supplementary Figure S4. Postpartum changes in milk IgG concentrations of mares in Group 1 and 2 during the study period.

A statistically significant difference in IgG concentration at 0 h postpartum was detected between Group 1 (107.98 ± 32.42 mg/mL) and Group 2 (38.12 ± 4.26 mg/mL) ($p < 0.05$). Similarly, a significant difference between groups persisted at 24h postpartum. However, no statistically significant differences were observed between groups from day 7 onward. In addition, a progressive decrease in milk IgG concentrations was observed throughout the postpartum period in both Group 1 and Group 2. Higher colostral IgG concentrations were detected in Group 1. However, in both groups, colostral IgG concentrations declined rapidly after 24h postpartum and remained at low levels thereafter in both groups.
