# Supplementary material for: Comparative Analysis of Mare Milk and Foal Faecal Microbiota in Kyrgyz Mare–Foal Pairs During the First 21 Days Postpartum
Source: Animals (Basel). 2026 Nov 1;16(21):3448. doi: 10.3390/ani16213448 (PMC13761987; doi:10.3390/ani16213448)
Supplement: Supplementary file 1 [file animals-16-03448-s001.zip › Supplementary Figure S5.pdf]

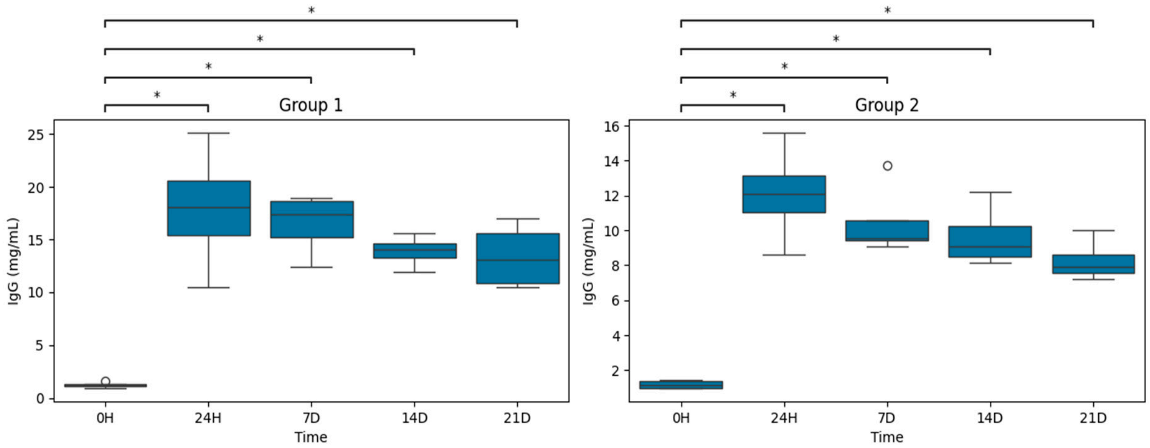

Supplementary Figure S5. Postpartum changes in serum IgG concentrations of foals in Group 1 and 2 during the study period.

Serum IgG concentration changes in foals from Group 1 and Group 2 during the postpartum period are presented. Serum IgG concentrations measured immediately after birth were very low in both groups, and no statistically significant difference was observed between groups at this time point ($p > 0.05$). At 24h postpartum, foals fed by Group 1 mares exhibited significantly higher serum IgG concentrations (18.86 ± 5.15 mg/mL) compared with foals in Group 2 (11.10 ± 2.91 mg/mL) ($p < 0.05$). Moreover, this statistically significant difference between Group 1 and Group 2 foals persisted throughout the postpartum period. In both groups, serum IgG concentrations were very low at 0 h postpartum. From 24 h onward, serum IgG levels increased markedly and remained elevated throughout the 21-day period. However, foals that received colostrum with lower IgG concentrations (Group 2) consistently exhibited lower serum IgG levels compared with Group 1 foals throughout the entire 21-day postpartum period.
