# Supplementary material for: Comparative Analysis of Mare Milk and Foal Faecal Microbiota in Kyrgyz Mare–Foal Pairs During the First 21 Days Postpartum
Source: Animals (Basel). 2026 Nov 1;16(21):3448. doi: 10.3390/ani16213448 (PMC13761987; doi:10.3390/ani16213448)
Supplement: Supplementary file 1 [file animals-16-03448-s001.zip › Supplementary Table S1.pdf]

Supplementary Table S1

Sample-level read counts during DADA2 processing for 40 mare milk and 39 foal faecal samples.

Mare milk

Sample	Time	Input	Filtered	Denoised	Merged	Non-chimeric	Retained (%)
M1_1	0 h	58,227	46,155	36,094	22,063	21,470	36.87
M1_2	24 h	88,018	75,079	67,798	58,461	56,266	63.93
M1_3	7 d	125,187	108,375	101,737	89,092	85,350	68.18
M1_4	14 d	95,389	81,286	75,818	67,715	65,325	68.48
M1_5	21 d	105,610	93,035	90,068	80,501	73,921	69.99
M2_1	0 h	174,004	139,500	125,416	101,486	77,720	44.67
M2_2	24 h	100,929	86,026	79,350	65,774	63,128	62.55
M2_3	7 d	181,866	145,348	118,946	83,621	77,564	42.65
M2_4	14 d	194,874	154,003	139,109	99,600	80,518	41.32
M2_5	21 d	347,530	283,890	265,007	215,050	163,036	46.91
M3_1	0 h	208,739	153,289	112,780	84,589	79,574	38.12
M3_2	24 h	89,843	75,472	66,624	55,428	54,154	60.28
M3_3	7 d	142,053	119,241	106,205	85,379	81,938	57.68
M3_4	14 d	105,027	92,499	88,322	80,759	78,016	74.28
M3_5	21 d	92,592	80,714	76,362	68,665	66,534	71.86
M4_1	0 h	70,082	56,268	46,636	35,877	34,823	49.69
M4_2	24 h	189,584	160,673	142,961	114,190	109,151	57.57
M4_3	7 d	1,209	1,064	828	698	686	56.74
M4_4	14 d	49,514	43,914	42,022	38,182	36,662	74.04
M4_5	21 d	17,614	15,204	13,640	12,264	11,933	67.75

Notes: M, mare milk; F, foal faeces. The first number identifies the mare–foal pair; the second denotes sampling time (1 = 0 h, 2 = 24 h, 3 = 7 d, 4 = 14 d, 5 = 21 d). Source-file prefixes S and D correspond to M and F, respectively; animal numbers are unchanged.

Supplementary Table S1 continued

Sample-level read counts during DADA2 processing.

Mare milk

Sample	Time	Input	Filtered	Denoised	Merged	Non-chimeric	Retained (%)
M5_1	0 h	43,961	38,354	36,197	33,047	32,102	73.02
M5_2	24 h	25,973	22,880	21,747	19,357	18,649	71.80
M5_3	7 d	60,174	50,317	48,601	42,551	37,151	61.74
M5_4	14 d	30,919	27,166	25,605	23,442	22,380	72.38
M5_5	21 d	45,831	39,014	34,867	29,814	28,681	62.58
M6_1	0 h	128,652	108,809	96,956	80,940	77,615	60.33
M6_2	24 h	169,896	144,160	132,906	110,510	105,511	62.10
M6_3	7 d	47,511	41,557	37,941	33,703	32,859	69.16
M6_4	14 d	152,615	129,413	118,372	99,063	93,565	61.31
M6_5	21 d	153,892	128,163	115,884	97,875	92,031	59.80
M7_1	0 h	108,281	92,866	86,523	77,955	71,434	65.97
M7_2	24 h	39,763	33,846	29,554	26,518	25,128	63.19
M7_3	7 d	84,651	70,667	59,350	48,392	45,647	53.92
M7_4	14 d	120,012	99,316	88,813	74,492	71,017	59.17
M7_5	21 d	101,568	83,294	71,467	61,128	58,705	57.80
M8_1	0 h	187,715	159,408	145,319	124,055	114,807	61.16
M8_2	24 h	102,758	80,307	62,738	47,908	46,410	45.16
M8_3	7 d	134,780	112,817	101,401	87,096	81,681	60.60
M8_4	14 d	110,710	94,432	84,076	71,605	66,817	60.35
M8_5	21 d	116,209	102,879	100,342	93,400	83,860	72.16

Supplementary Table S1 continued

Sample-level read counts during DADA2 processing.

Foal faeces

Sample	Time	Input	Filtered	Denoised	Merged	Non-chimeric	Retained (%)
F1_1	0 h	70,555	59,166	55,785	48,542	47,344	67.10
F1_2	24 h	65,726	58,759	57,992	56,703	54,068	82.26
F1_3	7 d	184,823	167,097	164,010	156,381	146,851	79.45
F1_4	14 d	110,682	98,472	95,802	88,958	86,485	78.14
F1_5	21 d	127,265	114,135	110,344	102,007	97,970	76.98
F2_1	0 h	213,706	190,685	183,299	161,013	147,988	69.25
F2_3	7 d	102,546	91,829	88,758	86,510	83,944	81.86
F2_4	14 d	228,221	201,904	195,237	178,026	169,731	74.37
F2_5	21 d	207,766	185,935	180,386	164,740	155,870	75.02
F3_1	0 h	109,650	91,210	80,424	64,752	59,546	54.31
F3_2	24 h	174,325	157,145	155,232	149,599	136,326	78.20
F3_3	7 d	72,117	64,638	64,030	61,857	55,945	77.58
F3_4	14 d	115,797	104,081	101,925	96,638	91,000	78.59
F3_5	21 d	151,893	136,511	133,149	123,794	117,008	77.03
F4_1	0 h	65,269	57,530	55,877	48,793	46,494	71.23
F4_2	24 h	107,177	96,775	95,188	92,038	84,416	78.76
F4_3	7 d	119,458	106,857	104,520	98,422	89,784	75.16
F4_4	14 d	168,861	149,887	144,619	127,359	119,611	70.83
F4_5	21 d	126,884	114,016	110,603	99,518	95,513	75.28
F5_1	0 h	199,922	180,687	176,076	161,110	148,833	74.45

Supplementary Table S1 continued

Sample-level read counts during DADA2 processing.

Foal faeces

Sample	Time	Input	Filtered	Denoised	Merged	Non-chimeric	Retained (%)
F5_2	24 h	87,357	75,485	70,394	59,253	57,120	65.39
F5_3	7 d	79,459	71,380	70,658	68,505	65,497	82.43
F5_4	14 d	125,087	111,758	109,650	103,901	93,537	74.78
F5_5	21 d	196,370	175,938	171,260	156,662	145,337	74.01
F6_1	0 h	124,961	113,081	111,613	108,198	102,997	82.42
F6_2	24 h	146,871	131,480	129,186	123,692	113,506	77.28
F6_3	7 d	195,384	175,092	169,875	152,626	144,421	73.92
F6_4	14 d	269,567	241,391	235,238	214,778	203,028	75.32
F6_5	21 d	118,027	102,782	96,267	81,925	78,449	66.47
F7_1	0 h	78,885	68,198	66,125	60,535	56,813	72.02
F7_2	24 h	87,078	77,979	77,098	74,470	67,871	77.94
F7_3	7 d	89,619	80,351	79,270	75,798	70,693	78.88
F7_4	14 d	79,350	71,672	69,916	64,885	62,156	78.33
F7_5	21 d	97,457	87,590	85,289	78,937	74,137	76.07
F8_1	0 h	45,998	39,749	37,475	30,188	29,058	63.17
F8_2	24 h	138,331	123,503	121,423	114,530	97,956	70.81
F8_3	7 d	126,731	112,901	110,353	101,629	87,846	69.32
F8_4	14 d	195,642	173,215	164,920	136,813	118,330	60.48
F8_5	21 d	67,471	59,808	57,390	50,361	47,204	69.96

Notes: Counts are taken from the DADA2 denoising-statistics exports. Retained (%) = non-chimeric reads / input reads × 100, rounded to two decimal places. Non-chimeric counts represent output after chimera removal, not counts after any subsequent taxonomic filtering or rarefaction. F2_2 (source ID D2_2) was not collected and is therefore absent. M4_3 (source ID S4_3) yielded 686 non-chimeric reads and did not meet a rarefaction depth of 2,000 reads; it is retained here to document processing of all collected samples.
